# Loeffler Syndrome in FIP1L1-PDGFRA-Positive Myeloid Neoplasm

**DOI:** 10.1016/j.jaccas.2025.106296

**Published:** 2025-12-11

**Authors:** Marta Alcalá Ramírez del Puerto, Carlos Sánchez Sánchez, Isabel Piñero Uribe, Cristóbal Urbano Carrillo, Daniel Gaitán Román

**Affiliations:** a Department of Cardiology, Hospital Regional Universitario de Málaga, Málaga, Spain; b Department of Cardiology, Hospital Santa Ana de Motril, Motril, Spain

**Keywords:** cancer, cardiac magnetic resonance, chronic heart failure, fibrosis, restrictive, thrombus

## Abstract

**Background:**

Eosinophilic myocarditis and restrictive cardiomyopathy (Loeffler syndrome) are rare but severe manifestations of hypereosinophilic syndromes, especially in myeloid/lymphoid neoplasms with tyrosine kinase gene rearrangements.

**Case Summary:**

A 40-year-old man presented with progressive dyspnea, constitutional symptoms, and marked eosinophilia. Imaging showed apical thrombi, restrictive physiology, and pericardial effusion. Bone marrow studies confirmed an FIP1L1-platelet-derived growth factor receptor α–positive myeloid/lymphoid neoplasm. Treatment with corticosteroids and imatinib led to clinical and echocardiographic improvement, eosinophil normalization, and molecular remission within 3 months.

**Discussion:**

This case illustrates eosinophilic cardiomyopathy secondary to a specific genetic neoplasm. Early recognition, multimodality cardiac imaging, and targeted therapy are essential to improve outcomes.

**Take-Home Messages:**

Cardiac involvement in hypereosinophilic syndromes requires multidisciplinary management combining cytoreductive therapy, anticoagulation when thrombus is present, and serial cardiac magnetic resonance. Testing for FIP1L1-platelet-derived growth factor receptor α is disease-defining and therapy-guiding given the marked response to imatinib.

## Visual Summary

**Figure:**
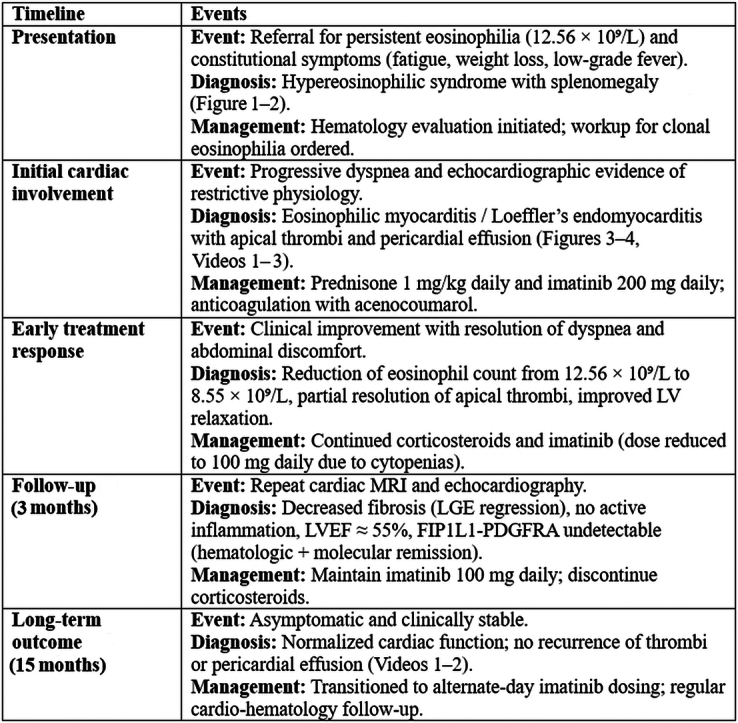

**Visual Summary:**
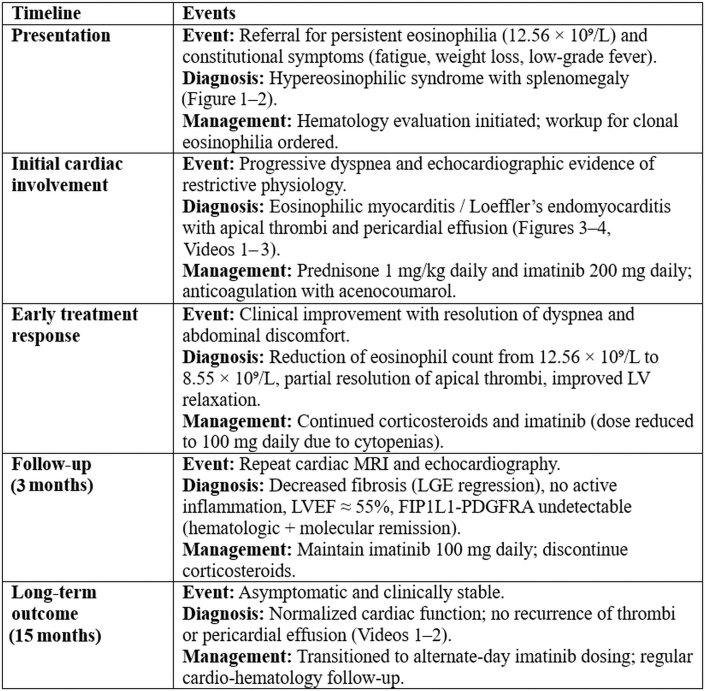
Clinical Course of PDGFRA-Positive Loeffler Syndrome

## History of Presentation

A 40-year-old man, active smoker, with no known medical history, was referred to the hematology clinic because of persistent eosinophilia identified on routine blood tests (absolute eosinophil count 12.56 × 10^9^/L). He reported several months of constitutional symptoms (asthenia, low-grade fever, and weight loss) and progressive left upper quadrant abdominal pain, which had intensified recently. He also experienced moderate exertional dyspnea but denied signs of overt heart failure. On physical examination, he had stable vital signs and significant splenomegaly (4 fingerbreadths below the costal margin) with tenderness on palpation. There were no signs of peripheral edema or jugular venous distension. Cardiopulmonary auscultation was unremarkable.

**Take-Home Messages**

• Cardiac involvement in hypereosinophilic syndromes requires multidisciplinary management combining cytoreductive therapy, anticoagulation when thrombus is present, and serial cardiac magnetic resonance.
• Testing for FIP1L1-platelet-derived growth factor receptor α is disease-defining and therapy-guiding given the marked response to imatinib.

## Past Medical History

The patient had no history of cardiovascular disease, allergies, autoimmune conditions, or parasitic infections. He had no regular medications and no significant family history. He denied illicit drug use and had no history of previous hospitalizations or surgeries. He worked as a warehouse assistant with no exposure to known cardiotoxins.

## Differential Diagnosis

The initial differential diagnosis for hypereosinophilia included:

• Secondary causes (eg, parasitic infection, allergic disorders, and solid tumors)
• Primary clonal eosinophilia (myeloproliferative neoplasms)
• Idiopathic hypereosinophilic syndrome (HES)^1^

Given the associated constitutional symptoms, splenomegaly, and high eosinophil count, a myeloproliferative disorder was strongly suspected. Cardiac involvement prompted consideration of eosinophilic myocarditis, including Loeffler endocarditis, which warranted immediate cardiologic evaluation.

## Investigations

Laboratory tests revealed leukocytosis (17,080/μL) with marked eosinophilia (73.5%, absolute count 12,560/μL) and elevated serum vitamin B12 (>2,000 pg/mL). No monoclonal protein was found. Polymerase chain reaction testing for BCR-ABL1 showed negative results.

Bone marrow biopsy and fluorescence in situ hybridization analysis revealed a heterozygous deletion in the CHIC2 region (4q12), consistent with the FIP1L1-platelet-derived growth factor receptor α (PDGFRA) fusion.

**Figure 1:**
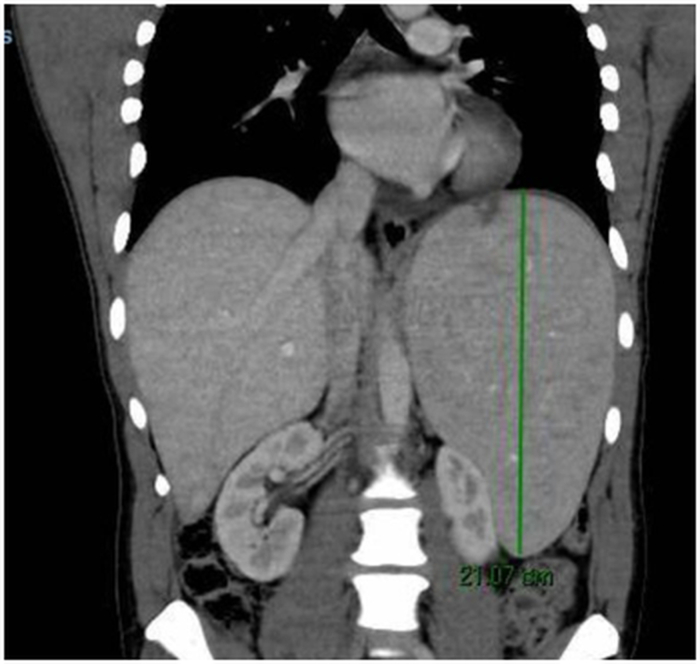
Computed Tomography With Marked Splenomegaly (21.1 cm) and No Focal Splenic Lesions

Abdominal ultrasound confirmed splenomegaly (18.8 cm) corroborated by computed tomography (Figure 1).

**Figure 2:**
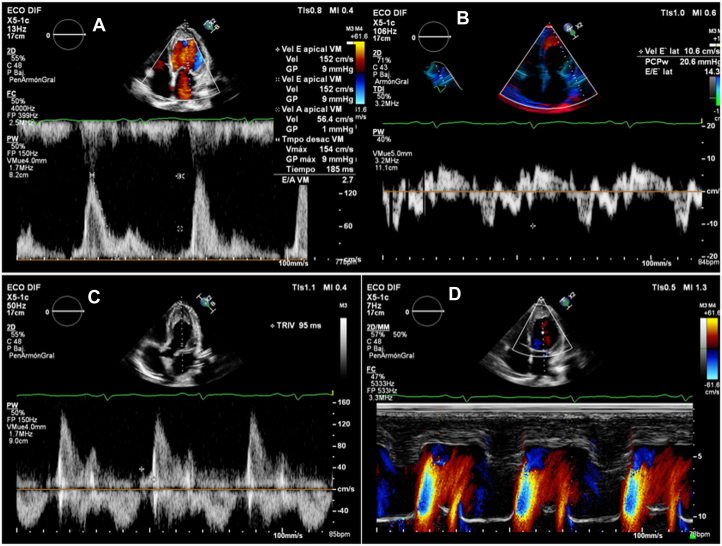
Echocardiographic Evidence of Restrictive Physiology in Eosinophilic Cardiomyopathy (A) Pulsed-wave Doppler at the mitral inflow showing a restrictive filling pattern with increased early diastolic velocity (E-wave 152 cm/s), reduced atrial contraction velocity (A-wave 56 cm/s), and a shortened deceleration time (185 ms), consistent with elevated left ventricular filling pressures (*E/A* = 2.7). (B) Tissue Doppler imaging (TDI) of the lateral mitral annulus demonstrating reduced early diastolic velocity (*E'* = 10.6 cm/s) and an elevated *E*/*E'* ratio (14.3), confirming increased filling pressures (PCPw 20.6 mm Hg). (C) Continuous-wave Doppler across the mitral valve showing a shortened isovolumic relaxation time (95 ms). (D) Color M-mode Doppler demonstrating rapid early diastolic transmitral flow propagation, characteristic of restrictive left ventricular physiology.

**Figure 3:**
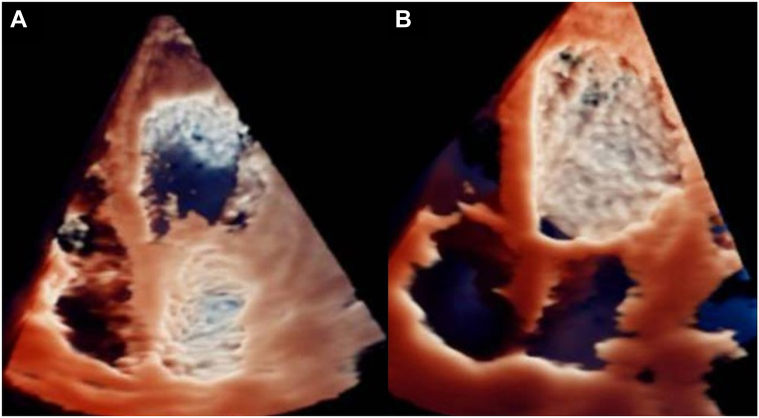
Baseline and Follow-Up Transthoracic Echocardiography (TrueVue) (A) Apical 4-chamber view showing obliteration of the left ventricular apex by intracavitary echogenic material, with reduced ventricular volumes. (B) Marked reduction of the intracavitary echogenic material after several months of imatinib therapy.

A transthoracic echocardiogram (TTE) showed biventricular involvement with a restrictive filling pattern, apical obliteration by echodense material, severe pericardial effusion without tamponade signs, and mild global hypokinesis (left ventricular ejection fraction approximately 50%) (Figure 2). Baseline echocardiographic findings with TrueVue rendering revealed left ventricular (LV) apical cavity obliteration by echogenic endomyocardial thickening and an intracavitary component (Figure 3A, Video 1). Follow-up TrueVue imaging after imatinib therapy showed re-expansion of the LV apex and a marked reduction of intracavitary echogenic material (Figure 3B, Video 2).

**Figure 4:**
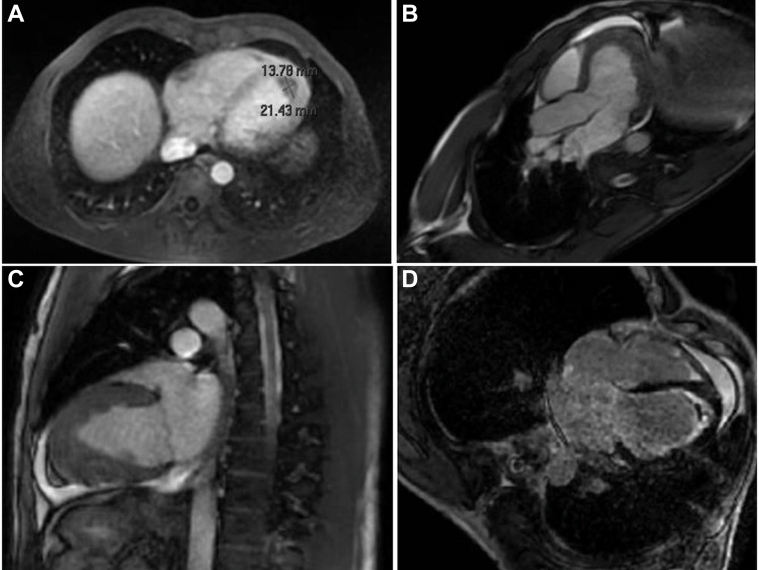
Baseline Cardiac Magnetic Resonance Images (A) Axial late gadolinium enhancement (LGE) image showing an apical thrombus in the left ventricle (LV) measuring 14 × 21 mm. (B and C) Cine SSFP sequences in the apical 3-chamber (B) and apical 2-chamber (C) views demonstrating an LV with apical thickening/obliteration due to intracavitary fibrotic endomyocardial tissue. (D) LGE study in the apical 4-chamber view showing circumferential subendocardial enhancement involving the apical and midventricular segments of the LV, with right ventricular involvement (apical thickening and a laminar subendocardial thrombus without enhancement) displaying the same enhancement pattern. SSFP = steady-state free precession.

Cardiac magnetic resonance (MRI) demonstrated subendocardial late gadolinium enhancement (LGE) consistent with fibrosis, laminar thrombi in both ventricular apices (LV: 21 × 14 mm), and mildly reduced systolic function (Figure 4). T2-weighted imaging suggested active myocardial inflammation. These findings were compatible with Loeffler endomyocarditis in the thrombotic/fibrotic stage.

## Management (Medical/Interventions)

The patient was diagnosed with eosinophilic restrictive cardiomyopathy (Loeffler syndrome) secondary to a myeloid/lymphoid neoplasm with tyrosine kinase gene rearrangement. Treatment was initiated with systemic corticosteroids (prednisone 1 mg/kg daily) to rapidly suppress eosinophilic infiltration and inflammation, followed by targeted molecular therapy with imatinib 200 mg daily.

Because of the development of cytopenias, the imatinib dose was adjusted to 100 mg daily.

Anticoagulation with acenocoumarol was initiated to address ventricular thrombi and prevent embolic events.

**Figure 5:**
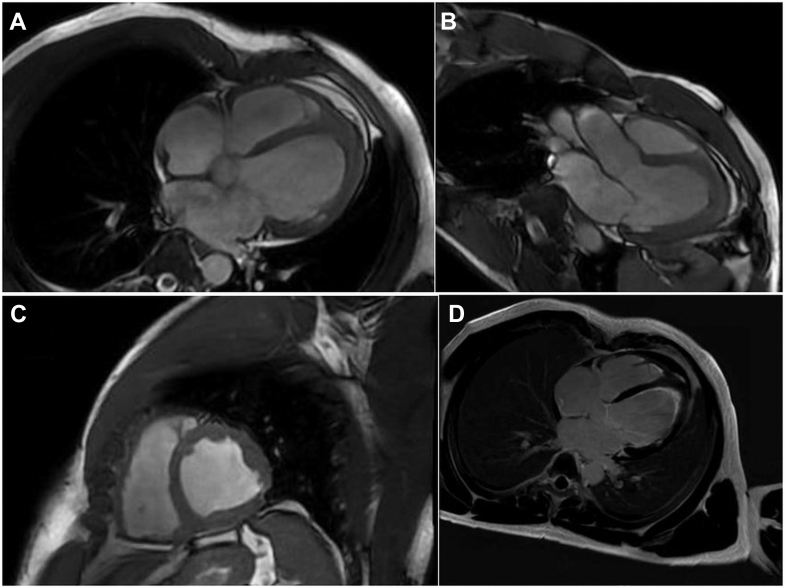
Follow-Up Cardiac Magnetic Resonance After Imatinib (A to C) Cine SSFP, apical 4-chamber (A), apical 3-chamber (B), and short-axis (C) views: nondilated left ventricle (LV) with residual apical thickening/obliteration; decrease in the size of the LV apical thrombus compared with the initial study. (D) Late gadolinium enhancement (apical 4-chamber): circumferential subendocardial enhancement at the apical level bilaterally (LV and right ventricle); additional subendocardial enhancement in the basal and midlateral LV wall, consistent with endomyocardial fibrosis. The LV apical thrombus appears laminar and smaller, without enhancement. SSFP = steady-state free precession.

Serial TTE was performed to assess the evolution of pericardial effusion, thrombi burden, and diastolic function (Videos 3 and 4). Cardiac magnetic resonance was repeated at 3 months to evaluate fibrosis and inflammation regression (Figures 4 and 5).

Multidisciplinary management was coordinated with cardiology, hematology, and radiology teams.

## Outcome and Follow-Up

Clinical improvement was observed within weeks, with resolution of dyspnea, asthenia, and abdominal discomfort. Serial blood tests demonstrated a rapid hematologic response, with the absolute eosinophil count decreasing from 12.56 × 10^9^/L at baseline to 8.55 × 10^9^/L after initiation of corticosteroid and imatinib therapy, and subsequently normalizing within a few weeks. Two-month echocardiography demonstrated partial resolution of apical thrombi, reduction in pericardial effusion, and improvement in ventricular relaxation parameters.

At 3 months, cardiac magnetic resonance showed decreased LGE extent, resolution of active inflammation, and improvement in systolic function (left ventricular ejection fraction approximately 55%) (Figure 5, Videos 3 and 4). Molecular testing confirmed that FIP1L1-PDGFRA was undetectable, indicating hematologic and molecular remission.

At 15 months, the patient remained asymptomatic with normalized cardiac function and was transitioned to alternate-day dosing of imatinib. Echocardiography showed no recurrence of thrombi or pericardial effusion (Videos 3 and 4).

## Discussion

Our patient presented with a nonspecific and insidious clinical picture that could initially have gone unnoticed. In this context, an appropriate physical examination (splenomegaly) and medical history (constitutional syndrome) were of special importance. Based on these signs and symptoms, along with the laboratory finding of eosinophilia and elevated vitamin B12, the suspicion was clear. The final diagnosis was established after bone marrow biopsy, revealing a myeloid/lymphoid neoplasm with eosinophilia and tyrosine kinase gene rearrangement.

In the myeloproliferative variant of HES, a rearrangement involving fusion of the FIP1L1 and PDGFRA genes is present in approximately 11% of cases. Greater disease remission has been reported with the use of tyrosine kinase inhibitors such as imatinib,^2^, ^3^, ^4^ making this clinical finding crucial and mandatory to screen for in these patients, as recommended in the current European LeukemiaNet and World Health Organization guidelines. According to a cohort of 135 patients, the incidence of blast phase, disease progression, and treatment response is markedly variable depending on the underlying fusion gene (higher complete remission rates with imatinib in patients with PDGFRA/platelet-derived growth factor receptor β fusion genes; more frequent and rapid progression to blast phase in FGFR1, JAK2, and ETV6::ABL1 fusion genes).^5^ In addition, cardiac involvement appears to be more common according to the literature in cases with this mutation, with a higher incidence of debilitating and potentially fatal cardiac compromise in the absence of therapy.^6^ For this reason, a TTE was requested for our patient, resulting in the diagnosis of Loeffler syndrome.

Eosinophil-mediated cardiac damage evolves through 3 stages, which may overlap and not necessarily occur sequentially:

• Acute necrotic stage (often asymptomatic with normal TTE findings)
• Thrombotic stage (characterized by thrombus formation along the damaged endocardium, with the potential risk of embolization of thrombotic material to distant sites)
• Fibrotic stage^7^ (with impaired cardiac function/heart failure due to restrictive cardiomyopathy and/or involvement of the chordae tendineae, leading to mitral and tricuspid regurgitation).

Echocardiography and cardiac magnetic resonance may reveal intracardiac thrombi or evidence of fibrosis, such as thickening of the posterior leaflet of the mitral valve or the posterior wall, LGE on cardiac magnetic resonance, and increased endomyocardial echodensity in fibrotic areas. Our patient presented with moderate exertional dyspnea, and TTE revealed restrictive cardiomyopathy with apical thrombus and apex obliteration. Several reports have demonstrated that contrast-enhanced cardiac magnetic resonance detects all stages and aspects of eosinophil-mediated cardiac damage, including the early stage of eosinophilic myocardial inflammation.^8^

Given the previously described FIP1L1-PDGFRA gene fusion, treatment with corticosteroids and imatinib was initiated, resulting in favorable progression and clinical remission. According to the existing literature, imatinib is generally well tolerated. However, patients with HES and cardiac involvement may be at risk of LV dysfunction and cardiogenic shock on initiation of imatinib therapy. The proposed mechanism likely involves massive release of eosinophil granule proteins with subsequent myocardial damage, although this has not been conclusively demonstrated. In some reported cases, heart failure was reversible with immediate administration of systemic glucocorticoids, intensive supportive care, and suspension of imatinib. Therefore, concomitant systemic glucocorticoid therapy (prednisone 1-2 mg/kg/day or equivalent) for 1 to 2 weeks is recommended when initiating imatinib in patients with any evidence of cardiac involvement.^9^ Although patients with PDGFRA/platelet-derived growth factor receptor β fusion genes generally respond well to imatinib, as previously mentioned, in cases of cardiac involvement, the outcome may vary despite adequate hematologic response, with cardiac disease being one of the main causes of morbidity and mortality, especially when advanced.^10^

The urgency of treatment and choice of therapy depend on the clinical presentation, laboratory findings, and results of mutational analysis, with a highly variable prognosis. Over time, earlier diagnosis, close clinical and echocardiographic monitoring of cardiac disease, and improved medical and surgical management of cardiac complications have increased the longevity of patients with HES. Likewise, targeted therapies—particularly the use of imatinib mesylate—are radically changing the historically grim prognosis of this disease.^7^^,^^11^

## Conclusions

Eosinophilic cardiomyopathy should be considered in patients with unexplained eosinophilia and cardiac symptoms. Early cardiac imaging, especially cardiac magnetic resonance, alongside genetic testing, is critical for accurate diagnosis. Timely initiation of corticosteroids and tyrosine kinase inhibitors such as imatinib can lead to rapid hematologic and cardiac improvement, even in advanced cases, and may prevent long-term sequelae through reversal of myocardial fibrosis and thrombotic complications.

### Data Availability

All data relevant to the case are included in the article and its figure legends. Additional details are available from the corresponding author upon reasonable request.

## Funding Support and Author Disclosures

The authors have reported that they have no relationships relevant to the contents of this paper to disclose.

## Appendix

Video 1 Baseline Transthoracic Echocardiography With TrueVue Rendering (Apical 4-Chamber View) Baseline imaging shows left ventricular apical obliteration by echogenic endomyocardial thickening with an intracavitary component, consistent with eosinophilic endomyocarditis.

Video 2 Follow-Up Transthoracic Echocardiography With TrueVue Rendering (Apical 4-Chamber View) After Imatinib Therapy Follow-up imaging demonstrates re-expansion of the left ventricular apex and a marked reduction of intracavitary echogenic material, consistent with structural improvement.

Video 3 Baseline Transthoracic Echocardiography (Apical 4-Chamber and Apical Long-Axis Views) Both views demonstrate left ventricular apical cavity obliteration by echogenic endomyocardial thickening with an intracavitary component (consistent with mural thrombus), associated with reduced ventricular volumes. These findings are consistent with eosinophilic endomyocarditis (Loeffler syndrome) in an FIP1L1-platelet-derived growth factor receptor α–positive myeloid neoplasm.

Video 4 Follow-Up Transthoracic Echocardiography (Apical 4-Chamber and Apical Long-Axis Views) After Several Months of Imatinib Therapy There is re-expansion of the left ventricular apex and a marked reduction of the intracavitary echogenic material, consistent with structural improvement. Global left ventricle systolic function remains mildly reduced.
